# Complexities of Socio-Labor Integration in Chile: Migrating Colombian Women’s Experiences

**DOI:** 10.3390/ijerph182111643

**Published:** 2021-11-05

**Authors:** Jimena Silva Segovia, Estefany Castillo Ravanal

**Affiliations:** 1 Postgraduate and Technology Transfer Directorate, Universidad de Tarapacá, Arica 1000000, Chile; 2 Faculty of Humanities, School of Psychology, Universidad Católica del Norte, Antofagasta 1240000, Chile; castilloravanale@gmail.com

**Keywords:** south-south migration, women, work, discrimination, Chile

## Abstract

The objective of the article is to understand Afro-Colombian women’s emotional experiences of the migratory process, and their labor insertion in Chilean territory. The Antofagasta region is one of the doors that connects Chile with its neighbors; at the same time, it is a national territory that is linked to important economic and human movements due to its mining activity. In the analysis of the data collected through of group and individual interviews conducted in the city of Antofagasta, we found experiences of xenophobia, labor abuse, discrimination, prejudices, and stereotypes articulated, along with the tendency of Chilean culture to value European traits over native Latin American traits.

## 1. Introduction

Human mobility among territories, conceptualized as migration, has occurred since the beginning of the first human settlements. As a process, it is not a new phenomenon or a contemporary product. With the exception of Africa and historically depopulated territories, there are no “native peoples”, since cultures are usually overcome by other cultures; this is why a nativist policy that imposes restrictions on the rights of those not born in a defined territory is not supported [1]. This process has somehow left its mark on the mentalities, affectivity, and practices of human societies, whether they give refuge to foreigners or strive to protect themselves from the others, the different, the travelers. This is a temporary set of attitudes that is more or less ambiguous, but subject to the situation and political interests of a particular group. There is practically no religion, set of historical norms or formal laws, folklore or family history that does not include a position and a discourse regarding migrating, migrants, and the phenomenon of migration as a whole. However, the increase in populations as a result of modernity, increased access to means of transportation, and the development of electronic communications and knowledge about promising places to settle, as well as the brutal increase in economic, political, social, and, increasingly, environmental pressures that force relocation have made the phenomenon of migration, under conditions that are contrary to human well-being and are increasingly frequent and complex, typical of this moment and of the last decades of the twentieth century.

Chepo et al. [2], in their analysis of international migration processes, report unprecedented figures as part of a complex global phenomenon. They indicated that in 2017, there were 258 million international migrants (3.3% of the world population) [3]. In recent years, there has been an increase of 15% in migratory flows to countries of the Organization for Economic Cooperation and Development (OECD); among these, Chile stands out, with an increase of 33% in new entries registered between 2015 and 2016 [4]. Currently, it is estimated that there are a total of 1,251,225 international migrants in the country (7.1% of the total population) [3]. The predominant migratory flow is south-south, distributed throughout countries such as Venezuela (23%), Peru (17.9%), Haiti (14.3%), and Colombia (11.7%) [5]. A total of 5.6% of the total migrant population in Chile (7000 people) corresponds to refugees and asylum seekers. In this study, all of the interviewees were economic migrants, considering that those who requested refuge had to travel to Santiago (the country’s capital) to undergo a complex process of recognition by the migration authorities, [6].

Among the phenomena derived from human mobility is the collision between groups that are established in a territory and those who are newly arrived, which results in harmful behaviors toward people who move. The question is, what motivates the unequal treatment, abuse, and exclusion that some social groups show toward others? What elements underlie the feeling of superiority that some groups (the inhabitants of a certain place) show over others (newcomers)?

Regarding the legal difficulties of migration processes, we know that the Migration Law in Chile, contained in Decree Law 1094 of 1975, which was in force until April 2021, originated during the period of military dictatorship. This, when considered within a logic of national security, gave excessive discretion to authorities, especially at the border, to control the entry and exit of people, a situation that is susceptible to being permeated by officials’ prejudices and stereotypes [7,8]. The content of this law established five types of temporary residence permits: student, subject to contract, temporary, resident with political asylum, or refugee. Refugee applications have been reviewed and are currently regulated by Law 21325 of 2021 [1].

One of the particularities of postmodern displacement is that it brings with it a strong presence of Afro-Latin women from different latitudes. María Emilia Tijoux (see Appendix A) [9] points out that the presence of migrant women in the country has generated an unsatisfied demand for domestic work. Thus, the need for child and elderly care among middle- and upper-class Chilean families creates a labor niche that has been covered by these Afro-Latin women. Within this nucleus, however, irregularities and human rights abuses are generated [9]. Since there are no strategies for domestic co-responsibility in terms of gender and no public policies regarding this matter exist when domestic workers (“nannies”) are hired, these gaps favor the prejudicial view that such work is for women with a lower socioeconomic status and a low educational level, even though in recent times, migrant women do not necessarily correspond to this stereotype, [1,10,11]. In line with the analysis of Tijoux [9], Millie Thayer points out that it usually happens that the south-south migrant population arrives in the country of destination to occupy a specific segment of the labor market, and Chile is not outside of this reality. The country’s citizens reject certain jobs for reasons related to salary or status, and these are typically precarious jobs that migrants usually fill. This population tends to occupy a subordinate position in the occupational structure, lower than that of the country’s citizens [12]. The preceding shows that many migrant workers do not have the option to access jobs with better working conditions, evidencing an intersection between migration and categories, such as gender, that deepen labor stratification [13].

The labor, economic, and political gaps that women already experience become more complex when they are intersected with the situation of migrants. In Chile, migration in recent years has increased from 45,000 people at the beginning of the 1990s [14], a period in which migratory flows to Chile intensified [15], to 1,492,522 as of 31 December 2019 [16]. Along with a transformation in volume, the feminization of migration has been observed worldwide [10,17,18] and in Latin America [19], and is accompanied by an increase in south-south migration, in which Chile appears to be one of the most frequent and desired destinations for migrants [20,21,22].

Regarding female participation in the labor market, as of 2017, approximately 43.4% of women nationwide were employed [23]. However, this rate is still much lower than the male employment rate of 60%, which has remained stable over time [24,25].

From a psycho-sociocultural perspective, we have observed that women are displaced not only by the political and economic problems they face daily in their countries of origin, but also by the greater demand of migrant women to insert themselves into certain types of work in the destination society [10]. For example, housework and cleaning, waitresses, caregivers of the elderly or children, among others, are very difficult to achieve for migrants who in some cases did not have paid jobs, and in others who were not paid enough to be self-employed. To understand how these works are inserted in the host society, gender is a category of analysis that makes visible power relations linked to the feminization of migration; it is a fundamental instrument for distinguishing the factors and processes that structure opportunities and rights—whether conventional or legal—for men and women in different societies, [24,25,26] Gender is linked to the scope of recognition, power, and status in any society. Gender operates in the structural organization of each culture by setting men and women in socially differentiated spaces (public or private), placing them in a hierarchical category (higher or lower) in the economic structure, and simultaneously imposing on them a social status (of greater or lesser prestige).From the perspective of the labor redistribution of migrant women, gender can be understood as one of the organizing principles in which they will be located in the economic structure of the receiving society (women, Afro-migrants, without a university education). From these classifications, the gaze of recognition is built, and with gender belonging, cultural patterns of interpretation are encoded in social status, which, on the one hand, makes it easier for them to earn more money, but remains precarious. [27,28].

The latest studies [28,29] note that Latin American women who move have 10 or more years of education, including secondary, technical, and higher education. This should allow them to expand their life plans and diversify their possibilities to achieve economic autonomy.

In the female experiences that will be analyzed in this article, a wide range of motivations and expectations can be observed, ranging from those that are focused on the family, to those that are defined by the women’s individual projects. In this research, we seek to answer the following questions: what does the experience of labor insertion in Chile mean for Afro-Colombian women integrated into the Antofagasta context? What emotional processes emerge in the face of different work interactions with Chilean employers?

### 1.1. Women Crossing Borders

According to different studies on the feminization of migration in the late 21st century, women have become advance subjects in contemporary migration networks. This trend has been observed in different Latin American countries [30,31,32]. Thus, they pave the way for other groups in their countries of origin, becoming the central nucleus of social networks that are progressively “transnationalized” [31,33,34]. The group of migrant women is articulated and organized into different national territories, thus reinventing the ways of being a family and the processes involved in socializing and caring for children [35,36]. Among their strategies for addressing problems when crossing international borders, migrant women expand their networks of contacts, express the emotional capacity to overcome adversity, and quickly incorporate knowledge of different cultures, which facilitates their displacement. Hence, we can consider them transnational subjects in the terms of Schiller et al. [37].

**Figure 1:**
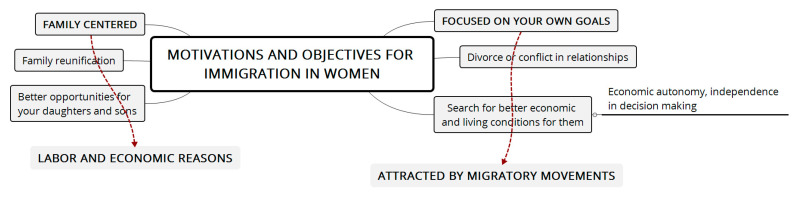
Motivations for the migration of Afro-Colombian women. Source: Own elaboration.

Regarding the motivations for displacement, economic needs are relevant. Migrant women have expectations, such as improving the quality of life of their children and saving and/or reunifying their family group; additionally, the need to be autonomous and independent is also observed. Many are divorced, are single mothers, or have unstable partners, (Figure 1 illustrates the trends found in this and other studies).

### 1.2. Motivations for the Migration

The following figure presents the motivations and objectives for the south-south migration of women, with two central focuses: family and personal goals.

## 2. Materials and Methods

This article reviews the selected results regarding the migration-labor axis from a broad qualitative study on the displacement of Afro-Colombian women to the Antofagasta region of Chile, and the psychosocial and emotional implications of their integration into the workplace.

In this article, we review the selected results regarding the migration-labor axis from a broad qualitative study on the displacement of Afro-Colombian women to the Antofagasta region of Chile, and the psychosocial and emotional implications of their integration into the workplace. The expanded research collects the experiences of men and women aged 18 to 60 years old (see Table 1), especially based on the motivations of migration and how they have been received in both work and study spaces, in the case of the youngest.

We have decided to focus this article on the women, migration, and work axis, since the Afro-Colombian women group in particular has suffered discrimination. We observe that the studied region, Antofagasta, has been culturally characterized by preserving a hierarchical and androcentric base in terms of work and gender, possibly due to its roots in mining work, in which men largely predominate.

This research was framed in a qualitative methodology, and the interpretative analyses were based on the experiences obtained from four conversation groups of six people each, and six in-depth interviews. The participants were Colombian women over 18 years of age who were included using the snowball technique. They were contacted in cultural centers and while waiting in lines at the Aliens and Immigration Affairs offices in Antofagasta, Chile. All of the participants signed an informed consent form permitting the use of their information and ensuring their anonymity, and the tool used in the study was evaluated and approved by the Ethics Committee of CONICYT (Comisión Nacional de Investigación Científica y Tecnológica.). Regarding educational level, the majority of the participants reported having completed high school. Regarding marital status, the participants were single, married, or partnered, and had one or more children. Additional interviews were also conducted with key informants who were workers at the regulatory institutions—Aliens and Immigration Affairs—and whose stories served as analytical counterpoints.

From the beginning, a good environment to conduct the interviews was challenging to obtain, as the women did not have much free time. These interviews were taken at the end of their work hours, in their free time, in long waiting lines (on the street) at immigration offices, and in places of recreation, such as parks or restaurants on holidays. We were willing to meet where they decided. We managed to meet for an hour and a half per session.

The interviews were organized based on a set of guide categories, such as (a) life story and reason for migration; (b) experience of the journey and arrival at destination; (c) relationship with residents of the contact countries; and (d) work experiences.

**Figure 2:**
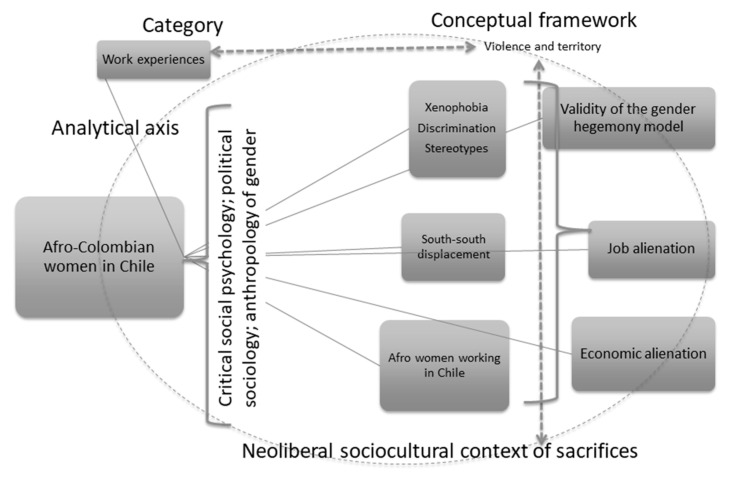
Global synthesis. The theoretical framework that supports the study. Source: Own elaboration.

All were recorded, after being authorized by the participants, and transcribed by the team. During all the meetings, field notes were taken, which were used as an informative basis for the first analysis. In the analyses, we used the grounded theory proposal, without the application of software, described in the Figure 2.

The experiences of the women interviewed from these groups gave us information that allowed us to saturate experiences, mainly in the aspects of xenophobia, discrimination, and gender violence. Among the products generated by this research, such as their stories and interviews, the research is synthesized in a documentary, elaborated based on script workshops carried out with the group of men and women. The final product was delivered to each of the participants as part of the return.

**Figure 3:**
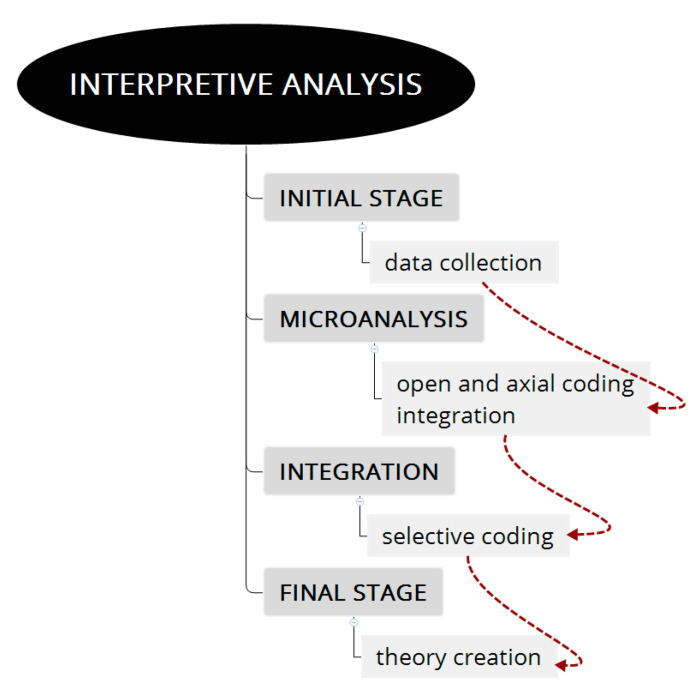
Diagram of the GT analysis process. Source: Own elaboration.

For the analysis of the corpus, we worked according to the grounded theory (GT) (see Figure 3) of Strauss and Corbin [38], applying a critical and gender perspective [39,40].

## 3. Synthesis of the Interpretative Analysis of the Findings

The following section presents the main categories of analysis that emerged in this study. These are organized under the Labor Situation of Afro-Colombian migrant women in northern Chile: Antofagasta axis, in which the following categories are identified: (a) work experiences and interpersonal relationships; (b) labor exploitation; and (c) self-employment.

### 3.1. Work Experiences and Interpersonal Relationships

According to United Nations [41], in the last four decades, there has been a gradual increase in the employment of women, which has modified women’s life options, situation, and gender position. The demand for women workers in the market is, in turn, linked to the association between domestic work and gender, which has stimulated the migration of women in different parts of the Western world. This movement is more strongly emphasized in the receiving countries, where the labor supply is permeated by functions associated with the female gender.

*A friend told me one day that, in Chile, they paid very well. “So, what do you work on there?” She told me doing cleaning in restaurants. “How much do they pay monthly?” Monthly, they pay her three million pesos! And I, oh, well, I’m going for six months! I’ll make money, and I’ll return to pay my debts. So, I went ahead, mortgaged my house for six million Chilean pesos, and I came here. When I arrived, how did my friend earn a little money? I am not good at that! I am old and I am embarrassed. In addition, in Chile, what I had to earn was 150,000 pesos. I’m telling you; I regret having come here! I had to work hard, to send the remittance, to pay the mortgage because otherwise, the bank would take my house. I paid it; now I am still working to send the remittance to my children, and for my own savings.*

(Aurelia, 60 years old)

In this sense, the massive hiring of immigrant women to perform domestic tasks and sex work accounts for the gender norms and stereotypes in the receiving countries. Likewise, the expectations that they bring with them from their country of origin—namely, that they will send remittances—can correspond to an increasing expansion of their role as main providers, breaking with the traditional position assigned to women-mothers-housewives, and with the collective imagination about the opportunities they might have in the receiving country.

*I thought I was going to earn like 100 million pesos (laughs), and when I arrived here, I did not earn anything. In addition, when I arrived here, yikes! This is a desert, it seemed to me, because there in Colombia, everything is green. I cried. I said to myself, “Why did I come here?”, but I have to stay! Because if I leave, I lose my house. (Addressing another person in the group) Did you think anything like that? Surely you did not think anything, that the change would be hard. No, you don’t think anything. Now you just have to deal with it.*

(Tolúa, 26 years old)

In the case of the mobilization of Colombian women, it is observed that, in their sociocultural context, family responsibilities have been reversed, as the women have assumed paid work outside the home, becoming heads of household to improve their family’s situation and their own survival. With these cultural modifications, the father has been displaced, and his figure as an absolute provider has become blurred. In contrast, when the participants arrived in Chile, they were forced into a place of subordinate domesticity that was subcontracted but paid. The whole process is linked to intercultural tensions, such as failures in migration laws, delays or high costs associated with processing or obtaining professional titles, documents or contracts that give them labor dignity and security.

*My mother is a very intelligent woman. She told me, “Daughter, why don’t you reconsider and leave, instead of staying here, waiting. You will not earn the same as you earned in Spain, but you will earn better than here with all the work you do”. So, it was those verbatim words from my mother that prompted me to make the decision. My fear was to leave my children because I have a 15-year-old girl, a 14-year-old boy, and a 7-year-old baby. I thought about protection: who is going to take care of them, you understand? That was my fear; also, fear for the older ones, that they would lose their way alone with my mother. She told me, “Trust, my daughter, that God, me and your sister are not going to let your children lose their way”. It was very painful to leave my family… I know that my mother is very important, my siblings, but to leave my children is to leave my life. Leaving them was the most difficult step I have ever taken.*

(Betty, 40 years old)

In this research, we analyzed testimonies of Colombian women, who describe the changes in the meaning of family well-being that are associated with gender advances: they consider themselves to be taking more risks than the Colombian men with whom they are involved, since they go out in search of ways to improve their lives. They also recognize gender differences in the meaning of family well-being. They point out that many men believe that stability, security, and taking few risks is the way out of their situations, while some women seek the stability of a better future for their children through risky actions, for example, by migrating. In doing so, they risk not only gender discrimination, but also ethnic discrimination in a country that retains strongly xenophobic cultural traits.

*People come here with their university studies, and they have not been able to get a job. If you are a university graduate, you will not go to a kitchen to wash dishes for 10,000 a day, because that is why you burned yourself out studying. I, who have not studied, nor the lady there who has not studied, nor the one here who has not studied, we can start washing dishes. It does not hurt us, because we do not have that gift of greatness; we could not go study, and so we start washing dishes.*

(Ovia, 33 years old)

According to Tijoux (2007), the presence of the Afro-Latin population in the public sphere questions the normative order based on the belief that, in Chile, there are no people of African descent, and the fiction that this country is a mostly “whitened” society, which makes people of other skin tones, native peoples, and those of mixed Andean races invisible. By ignoring this characterization of the Chilean population, the dark-skinned foreigner shakes up what the population is trying to deny: ethnic/racial origin.

*Here, there are many girls in the nightclubs; they have to make a living. It is because of the way they are treated at work; they are paid very little, they are humiliated a lot, because of their brown skin. So, what they do, they do not have much to think about; they have to earn to survive, to send home. And so, the first way out they saw was that: the nightclub.*

(Cris, 30 years old)

In addition, the fear that the “other” will have relationships with Chilean men or women, have children, and “contaminate the blood” is exacerbated. There is a need to whiten bodies to make them more akin to European bodies, those of a “dominant civilization”. Thus, according to cultural studies, a hidden racism is inscribed as a prominent feature of Chilean identity [42] (p. 231), a product of the historical reconfiguration of society.

### 3.2. Labor Exploitation

According to various accounts, labor exploitation and violations of current labor laws operate together with discrimination. In the Antofagasta region, there are sectors in which migrant workers are concentrated, such as domestic services, industrial cleaning, and working in the kitchens and waiting on tables in restaurants. In these positions, migrant women are preferred by employers, since they adapt to demands to meet their survival needs and the family commitments they have assumed. As a result, they are required or exposed to excessive work hours with low salaries and under worse conditions than Chileans who work in the same positions [10,27].

*I had to work for Doña Rosa even on Sundays—did you hear that? —For 7000 pesos (US $9.45) per day that she was going to pay me but then never did; I ended up working for free.* [And you cannot report her?] *No; the money she said she would pay me; she says she already paid it to me. There is no point in confronting her.*

*And if I do other things, it’s bad. If I go to work at a nightclub, it’s bad. If I am a street vendor, too. What do I do? Because if I go to work at a store, they pay me 8000 (US $10) for washing dishes all day.*

*Not even for 10,000 pesos (US $13). But for 10,000 pesos, I will not work for anyone! No, I’m not going to break my back working. Start at eight in the morning, leave at six or seven in the afternoon. Breaking my back all day for that money? No!*

(Tina, 43 years old)

It should be noted that discrimination is applied equally for most female immigrants.

*Well, on several occasions, I have been standing like this, taking the car, and one day some people called us “black”, and I… I no longer know how God forgives. Get out of my country. I was with my daughter; we came from church. They do not know how we are (…) because some compatriots work in those places (of prostitution). We are not all [like that]. They believe that we are all equal, and we are all discriminated against for the same reason, we women, because we are Colombian (…) whether we are white or black, that all Colombians come to prostitute themselves, but no, it is not like that. Because we have some people who come to work honestly, right? For them to know us, they have to look at people… those who have come to prostitute themselves, and those who are honest, who work honestly.*

(Yiam, 55 years old)

These prejudices and ethnic stereotypes fall mainly on Afro-Colombian women, who express feelings of being assaulted because of who they are. These conditions are not sufficient to avoid the humiliation and violence that they continue to experience during the process of labor integration. The experiences of Daysi and Mary illustrate this point:

*Getting a job is easier for women than for men in the sense that the trip to another country is easier than for men because women find work easier than men. He (her partner) does not leave Colombia. He has his son; well, he plays it safe, as they say there (to keep an ecologically stable place). He has a monthly pension. So, he is not going to retire from there to receive that money. I seek to improve my life.*

(Daysi, 22 years old)

*Yes, you do not turn your nose up at it; there is more work for a woman because we can get involved in washing dishes, as a waitress in a hotel, taking care of children, washing bathrooms, whatever. This is not the case for men.*

(Mary, 27 years old)

### 3.3. Being Self-Employed

Some participants compared the situations of salaried work and self-employment. Their work is related to the informal sale of food. They risk being detained daily, along with fines and the loss of their investment if it is confiscated. The independence of working on their own, although it enables them to avoid being mistreated by an employer, brings with it vulnerability. For example, these informal independent workers do not have access to social assistance services (health benefits, pensions, housing loans, etc.) due to the lack of a contract. However, they achieve independence, which gives them feelings of satisfaction and dignity.

*I have all my credentials as an instructor, as a trainer, as a teacher; I have all my credentials, and I also have my degree, which I studied for in Colombia, in Cali. Terra Instituto de Cali; I finished. I have not been able to get a good job here.*

*I am a hairdresser. I studied manicures, pedicures, cutting and styling. All those things. I do have the papers for that.* [And what do you work on here?] *I sell potatoes.*

*She does that like that other girl who sells rice; she goes around with a supermarket cart. The police bother her at all hours because they know she is selling. One day, they threw away her food. They took away the breaded chickens, and the rice was thrown away. So, she lost her money, her invested money. Thank God, they have never taken it from me.*

(Darlis, 41 years old)

Self-employment offers the women the advantages of managing their time and acquiring economic power and greater profits. However, they must accept and face the associated risks; thus, they are located in agency and in opposition to labor subordination. On the one hand, they are aware of the benefits they would have if their work were organized according to municipal and health regulations, but on the other, they avoid unequal interactions with employers who do not respect their labor rights.

*So, I work better with mine; I sell potatoes, and the money I earn is mine.*

*When I arrived, I worked in the Santa Isabel supermarket doing cleaning. They paid me 144,000 pesos. Imagine, what was that enough for? For nothing. (Selling potatoes), I earn more: my money to pay my rent, send to my children and survive.*

(Estrellita, 35 years old)

As another point of tension, they must face the demands from immigration services, which indicate that they should not work because they do not meet the required length of stay in Chile. In this situation are women and their partners who have not been able to legalize their residence, but must nonetheless find a way to meet their day-to-day survival needs. Not only must they engage in informal work, but their lives are precarious, and they are trapped in feelings of anguish, hopelessness, anger, and injustice.

*The carabinieri cannot see me selling because [if they do], they chase you. Because you do not have permission to sell. However, I have been to the municipality several times, and they do not give permission to sell street food; they do not give permission. I have the final visa. What they take from you, they eat it; sometimes, they throw it away. That food has never hurt anyone. If I had hurt someone, then no, do not let us sell it. However, it is a very clean thing, very hygienic and all those things. This past week, the inspectors of the municipality took a part of my earnings. I have to go from Monday to Tuesday to pay there. I am looking for a venue, and I cannot find one. They are very expensive venues, a million and something. I cannot pay a million and something, no.*

*There was a girl who was pregnant, who was already ready; on these days, she gave birth here, in the hospital. Anderson (the father) was deported; he is still there in Colombia, and the woman stayed here. Well, she had her mother here, and she gave birth; the mother helped her, [and] they deported him. She was appealing and appealing, and they had not answered anything. She has sent papers because he had already paid for his resident card.*

(Salomé, 43 years old)

## 4. Remittances Resulting from Work

According to Benito [43], remittances increase family income, and constitute a source of poverty reduction. In developing countries, remittances provide support for family members who do not migrate, and are invested in education, health, and housing. This makes it possible to access better levels of education and health, and reduces the social vulnerability of family members, especially women and children; furthermore, it allows the children to achieve greater cultural capital in the long term, which can balance the deficiencies in the countries of origin caused by emigration. In 2006, remittances comprised 2.9% of Colombia’s GDP (The Gross Domestic Product). In this way, the remittances sent by the interviewees improved the quality of life of their families in Colombia and increased their opportunities to study and have a better future. This, to some extent, compensates for the departure of Colombian nationals.

*I tell you that I live in the Pinares sector, Iquique Street. I have to pay remittances for my son and daughter, who are over there, and here, I have to pay 90,000 pesos a month in rent. An apartment, it has a bathroom. There is the kitchen; it is where we put the bed at night. The whole sky can be seen above, all hollow, all broken. When it rained, everything got wet. They charge the rent and do not fix anything for you. I live very badly. I am looking for an apartment or a little house. That neighborhood where I live is like the neighborhood of El Chavo—very ugly, very horrible house. Many people, many shacks.*

(Daysi, 22 years old)

*Those of us who live there are almost all Colombians. There are Colombians, Peruvians, and Chileans. I have to live like this because most of what I earn, I send to my children and my mother, who takes care of them. Here where I am is the neighborhood of El Chavo. There are like 23 rooms, but we all get along well. Yes, the house is very large; we all live well there. The little house is ugly, they have it all ugly, but [it is] very, very quiet there. There are like four bathrooms shared for everything. My room is about this size, from here to there, the size of the living room; large. The lady pays for everything. That is, if she disconnects the light in one room, she only disconnects it there, not in the other rooms. So, she knows who owes… the lady pays for her water and electricity.*

(Tolúa, 26 years old)

## 5. Discussion

In recent decades, countries receiving migrant women have seen an increase in the commercialization of domestic work. In recent years, Chile has become an emblematic example of a country that has a deficit of care workers and that, to meet these needs, has become a recipient of migrant workers to take over the work of social reproduction, which was previously occupied by mothers who were exclusively dedicated to domestic work.

Studies from the last decade in Latin America [10,44,45] have shown that the feminization of south-south migration is not solely the result of economic problems in the society of origin or the gaps in certain sectors (childcare, elderly care, home care, etc.) of the destination society. In Latin America, this mobility of women can also be understood as part of the normative changes in the gender order through which women have been constructing subjectivity in contemporary societies. Acosta [44] states that migratory models among Latin American migrant women are highly diverse, and range from migration focused on the family to migration defined according to the individual expectations of the migrant woman [44,45].

Regarding the valuation of migrant women’s labor potential, Fraser [46] links tensions to the field of recognition, arguing that gender refers to a two-dimensional social differentiation, a hybrid category that is simultaneously involved in the economic structure and the status of society. From the perspective of redistribution, gender can be approached as an organizing principle of the economic structure of society, while from the perspective of recognition, gender encodes cultural patterns of interpretation into social status. With Honneth [47], we can review the idea that the cultural valuation of specific capacities for success leads to the social demarcation of professions, and addresses the relationship between recognition and gender, evidencing the existence of prejudices regarding the capacities of women in the social construction of labor and professional fields. According to the author, in a hierarchy of social status, work activities that are predominantly practiced by women will tend to have decreased prestige and recognition, while those predominantly practiced by men will tend to have increased prestige and recognition, which explains the undervaluation of work that is primarily performed by women.

This situation has revived the exploited labor situation that had been disappearing in previous decades due to the massive entry of women into education, and their access to greater economic power [48,49]. Women had expanded their citizenship rights, and there was a clear resistance to subordination [39,40,41,42,43,44,45,46,47,48,49,50]. However, the emerging increase in female migration has unleashed a proliferation of precarious jobs, social discredit, low wages, lack of regulation, and invisibility for women who migrate [10,51], who, in turn, develop feelings of hopelessness and anguish for both themselves and their children.

The precariousness of migrant women is intertwined with institutional deficiencies, prejudices, and stereotypes; inefficient regulations, as well as a weak political will to improve immigration processes. Migrant women are exposed to work without a contract, abuse by employers, and the risk of labor exploitation. These experiences are also associated, in many cases, with feelings of insecurity caused by procedural factors because of delays between decisions and the issuing of official papers that result in missed deadlines.

It was observed that some women do not report abuses due to ignorance of their options, lack of accurate information, and emotional factors, such as the fear that they, and not their employers, will be sanctioned; therefore, labor abuse in the private spaces of the power relationship between employee and employer is invisible. It was observed that among employers that exploit Afro-Colombian women, there are Chilean men and women who take advantage of migrant women’s desperation in relation to labor insertion and the low level of prestige that female domestic work has, which leads to an association with low pay and decreased respect for and symbolic value of the person who performs it.

These attitudes, which are intertwined with xenophobia, are found in all socioeconomic levels of the population, and feed a perverse circle, falling as they do on both migrant women and on Chilean women who perform the same work. Women in both groups are hindered in accessing economic capital; they face restricted opportunities to incorporate cultural capital, and are limited and trapped in fields with less symbolic capital because they are migrant women, of African descent, and are considered displaced (however, this is less true for Chilean women, who have achieved greater protection of their rights, and therefore are exposed to less discrimination than migrant women).

Some limitations of the study can be found in that the interviewees belong to a medium-low socioeconomic level where the research was focused, leaving out the work experiences of women with more resources. Regarding the experience of researching in this field, the team allowed it to open a comprehensive line of community work, guiding collaboration groups with greater clarity on the needs of the migrant population, such as prevention of violence, sexual body self-care, among others. We received very significant feedback that was recorded in the documentary produced with a joint script.

**Figure 4:**
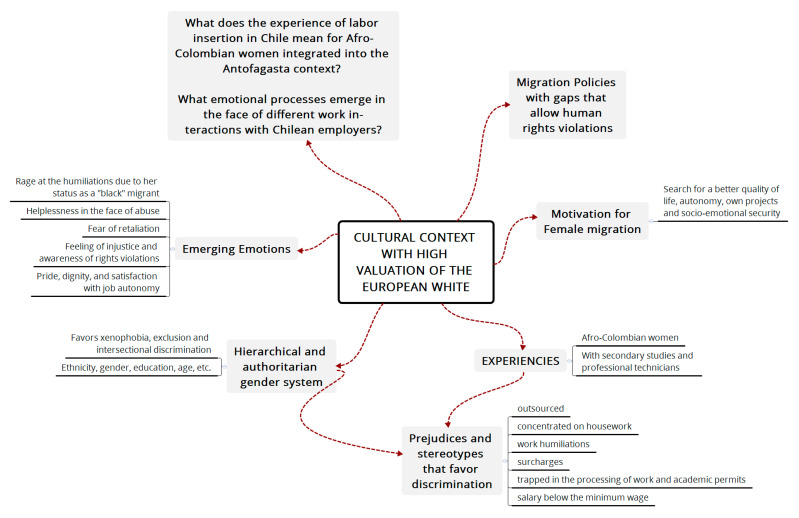
Emerging Analysis Model. Source: Own elaboration.

Figure 4 below shows a synthesis of the analytical process that helped to answer the initial questions from an intersectional and gender perspective, articulating the processes of insertion of Colombian women into an androcentric context that hypervalues whitened skin and its European ethnic associations over Caribbean and Latin American appearances. These predominant cultural characteristics in the country of arrival (Chile), with respect to Afro-Colombian women, generate attitudes laden with symbolic violence, which makes it difficult for the participating women to achieve dignified labor insertion with respect to their basic human rights. In contrast, their arrival in Chile was marked by tension, and they have experienced situations that have generated painful emotions (illustrated in Figure 3).

### Emerging Emotions

**Figure 5:**
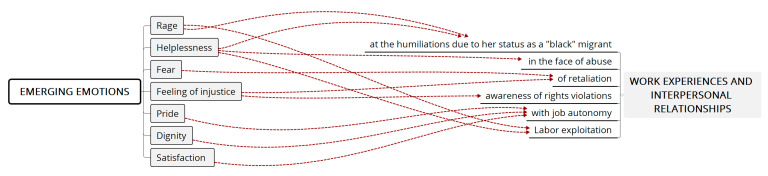
Emerging emotions. Source: Own elaboration.

Emotional expression comprises a symbolic structure that is articulated from the dynamics between the individual’s experience in daily life and the normative and gender order that have has culturally constructed to regulate these experiences (see Figure 5). That is, as we have explained in the analysis of the narrated experiences, these processes are required conditions for understanding the sources of these women’s emotions, associating their emotional experiences with the framework of the culture of origin and the culture of arrival and, as stated at beginning the of this article, identifying the ways in which their emotions are embodied to serve as a link between individual experience and the subject’s *Verstehen* of the reality that they discover.

In this context, we find that many women travel to Chile with high expectations. Upon arrival, they are faced with a reality that generates contradictory emotions, such as feelings of regret that are aggravated by the impossibility of returning to their country of origin due to the economic risks that they assumed when they migrated. The interviewees expressed longing for their country of origin, and constant comparisons between Chile and Colombia characterized by a greater appreciation of their native country. According to Julve et al. [52], the existence of a positive memory of the country of origin arises from mourning for the loss of proximity to customs, land, and contact with their own ethnic group, among other factors that influence perceptions regarding the place of origin and the receiving country. In this sense, Restrepo [53] points out that in Spain, immigrant women also express generalized feelings of nostalgia for the better housing conditions in Colombia. A more favorable memory of their past life reflects the pain of being uprooted. In this sense, housing not only represents a change in infrastructure conditions, for better or for worse, but also a void with respect to social and cultural relations [52].

Chile must address immigration in two fundamental ways. First, it must define how it will face migratory flows from other countries and clarify what type of border opening will be the most appropriate for the coming years. Second, the government should specify how it will manage its internal policies or propose the creation of new policies regarding the treatment of undocumented immigrants who enter and settle in Chile [54], especially given the repercussions that such regulations have on the beliefs of nationals. In addition, the adopted decisions must be consistent with agreements that have already been signed with the constitutional regulations, and the way in which they are expressed in the education of Chileans. As the interviewees reported, there is a greater emphasis on the preparation of intellectuals than on implementing integration policies.

As Larraín points out that, when “national identity is not defined as an unchangeable essence, but rather as a permanent historical process of construction and reconstruction of the ‘imagined community’ that is the nation, then the alterations that occur in its constituent elements do not necessarily imply that national identity has been lost, but rather that it has changed, that it is being built” [42]. The reconstruction of national identity must incorporate the presence of people of other nationalities who have come to stay, and to increase the national cultural heritage, enriching it with greater variety.

## Figures and Tables

**Table 1:** Afro-Colombian women participating in the research.

No. of Participants	Code	Age	Occupation		City of Origin (in Colombia)
			Minor, Unskilled, Day Laborer, Domestic Service	Unskilled Occasional, and Informal Work	
1	Betty	40		√	Antioquia
2	Tolúa	26		√	Bogotá
3	Aurelia	60	√		Cali
4	Ovia	33	√		Cerritos
5	Cris	30	√		Buenaventura
6	Tina	43	√		Buenaventura
7	Yiam	55			Bolívar
8	Darlis	41		√	Nariño
9	Estrellita	35		√	Bogotá
10	Salomé	43	√		Risaralda
11	Daysi	22	√		Valle del Cauca
12	Mary	27	√		Valle del Cauca

## Data Availability

The data supporting the findings of this study are available on re-quest from the corresponding author (J.S.S., FONDECYT 1180079). The data are not publicly available, due to their containing information that could compromise the privacy of research participants.

## Appendix A

María Emilia Tijoux

We consider the historical sociological analyses of Tijoux and Stefoni, who add depth to our analysis of the problem under study, incorporating new edges in the framework (María Emilia Tijoux, Sociologist and Doctor in Sociology at the Paris 8 University. She is an academic and researcher in the Department of Sociology at the Faculty of Social Sciences and Coordinator of the Doctorate in Social Sciences at the University of Chile. She coordinates the Research Center Sociology of the body and emotions and is Director of the Actuel Marx Interventions Magazine. Her research addresses immigration in recent decades, xenophobia, racism, sexualization and racialization of immigrants and their dehumanization processes. She has just edited the book “Racism in Chile: skin as a mark of immigration”.
